# Ophthalmic Paracoccidioidomycosis

**DOI:** 10.4269/ajtmh.17-0613

**Published:** 2017-12-06

**Authors:** Juan Cataño, Alexander Salinas

**Affiliations:** 1 Infectious Diseases Section, Internal Medicine Department, University of Antioquia School of Medicine, Medellín, Colombia;; 2 Infectious Diseases Section, University of Antioquia School of Medicine, Medellín, Colombia

**Figure 1.**
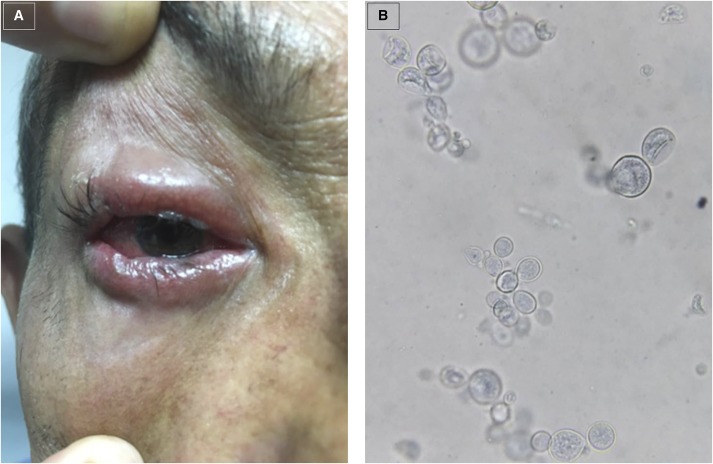
(**A**) Severe right-eye edema and purulent secretion consistent with blepharitis. (**B**) Eyelid biopsy showing multiple, narrow base, budding yeast cells of *Paracoccidioides brasiliensis* on lactophenol blue staining. This figure appears in color at www.ajtmh.org.

A 51-year-old man, with no remarkable medical history besides being a chronic smoker, had been working his entire life as a farmer in a rural area of Colombia (Bajo Cauca-Antioquia). He presented to the Infectious Diseases out-patient clinic with 6 months of progressive right eyelid edema, with purulent secretion but without any signs of periorbital cellulitis and a normal visual acuity, for which he had been treated with many different eye drops (steroid and antibiotic eye drops) without any significant clinical improvement. His only complaint was about subjective fevers and 5-Kg weight loss. Physical examination demonstrated that he was malnourished, with normal vital signs and had a severe right eye blepharitis (Figure 1A). The remainder of the exam did not show oral, chest, or abdominal findings. Then an eyelid biopsy was performed, showing multiple, narrow base, budding yeast cells (the “steering wheels”) of *Paracoccidioides brasiliensis* on lactophenol blue staining (Figure 1B). Culture on Saboreaud’s medium confirmed the mycological diagnosis. Treatment with oral itraconazole solution led to significant clinical improvement without any relapse on 6-months follow-up.

Paracoccidioidomycosis (or South American blastomycosis) is a systemic mycosis of high prevalence in Latin America, caused by dimorphic fungus *P. brasiliensis*. It has different clinical forms and may affect any organ or system, but the ophthalmic involvement like the one seen in this case is rare, and when it occurs, it is usually secondary to primary ocular infection spreading by contiguity.^1^ Isolated active lesions are usually diagnosed as malignant tumors, and cicatricial changes are characterized by a high degree of fibrosis.^2^ If not treated, the mycosis can destroy the eyelid, but most of the cases can be treated with triazoles, being itraconazole considered the treatment of choice in mild-to-moderate cases, and amphotericin B is recommended only for severe cases.^3^
